# Induction therapy with bortezomib and dexamethasone followed by autologous stem cell transplantation versus autologous stem cell transplantation alone in the treatment of renal AL amyloidosis: a randomized controlled trial

**DOI:** 10.1186/1741-7015-12-2

**Published:** 2014-01-06

**Authors:** Xianghua Huang, Qingwen Wang, Wencui Chen, Caihong Zeng, Zhaohong Chen, Dehua Gong, Haitao Zhang, Zhihong Liu

**Affiliations:** 1 Research Institute of Nephrology, Jinling Hospital, Nanjing University School of Medicine, Nanjing 210002, China

**Keywords:** AL amyloidosis, Bortezomib, Autologous stem cell transplantation

## Abstract

**Background:**

Although the use of bortezomib alone and in combination with steroids has shown efficacy in AL amyloidosis, its role in combination with high-dose melphalan and autologous stem cell transplantation (HDM/SCT) is unknown. In this study, we evaluated bortezomib in combination with dexamethasone (BD) for induction chemotherapy prior to HDM/SCT.

**Methods:**

This was a single-center, prospective, randomized controlled trial comparing induction therapy consisting of two BD cycles followed by HDM/SCT (BD + HDM/SCT) with HDM/SCT alone in the treatment of patients with newly diagnosed AL amyloidosis. The hematological and organ responses of the patients were assessed every three months post HDM/SCT.

**Results:**

Fifty-six patients newly diagnosed with renal (100%), cardiac (57.1%), liver (7.1%), or nervous system (8.9%) AL amyloidosis were enrolled in this study; 28 patients were assigned to each arm. Two patients died within 100 days of HDM/SCT (3.6% treatment-related mortality). The overall hematologic response rates in the BD + HDM/SCT arm and HDM/SCT arm at three, six and twelve months were 78.5% versus 50%, 82.1% versus 53.5% and 85.7% versus 53.5%, respectively. In the BD + HDM/SCT arm, 15 (53.5%) patients achieved a hematologic response after BD and before HDM/SCT. An intention-to-treat analysis revealed a higher rate of complete remission in the BD + HDM/SCT arm at both 12 and 24 months (67.9% and 70%, respectively) than with the HDM/SCT-only therapy (35.7% and 35%, respectively, *P* = 0.03). After a median follow-up of 28 months, the survival rates at 24 months post-treatment start were 95.0% in the BD + HDM/SCT group and 69.4% in the HDM/SCT alone group (*P* = 0.03).

**Conclusions:**

Our preliminary data suggest that the outcome of treating AL amyloidosis with BD induction and HDM/SCT was superior to the outcome of the HDM/SCT treatment alone.

**Trial registration:**

This trial has been registered at clinicaltrials.gov with the number NCT01998503.

## Background

AL amyloidosis is the most common form of systemic amyloidosis, and it arises from the production of monoclonal free light chains (FLCs) by a pathological plasma cell clone [1]. The deposition of insoluble amyloid fibrils in vital organs, such as the heart, kidney, liver, and nerves, can lead to progressive organ dysfunction and death [2]. AL amyloidosis is often devastating, and the median survival rate of untreated patients with this lethal disease is only 10 to 14 months from diagnosis [3]. The treatment for AL amyloidosis is aimed at reducing FLCs by eradicating the plasma cells [4] that produce them, although the options currently available for this treatment are limited [5].

AL amyloidosis treatments are based on existing multiple myeloma therapies. Melphalan (MEL) and prednisone administration was the first effective regimen developed for AL amyloidosis; however, the treatment responses are typically slow and rarely result in complete remission [6,7]. An Italian study combining MEL with high-dose dexamethasone (MDex) noted an impressive hematologic response rate of 67% [8] and long-term remissions in AL amyloidosis [9], and MDex is still considered a standard for non-study, non-transplant intervention because of its low toxicity profile [10]. Alternatively, intensive therapy with high-dose MEL and autologous stem cell transplantation (HDM/SCT) is effective in AL amyloidosis and can offer durable remission in some patients [11,12]. However, only 25% of affected patients are eligible for this approach [13,14], and the treatment-related mortality (TRM) with HDM/SCT is high [11,15,16]. Accordingly, it is necessary to identify modifications to the HDM/SCT procedure that could improve outcomes in patients with AL amyloidosis.

Novel drugs, including thalidomide [17,18], lenalidomide [19] and bortezomib [20] (alone or in combination with dexamethasone), have recently proven to be effective in the non-transplantation setting. Bortezomib is a reversible proteasome inhibitor that has shown significant activity in patients with multiple myeloma [21,22]. Indeed, several studies have confirmed that bortezomib combined with dexamethasone (BD) is an active and fast-acting regimen for AL amyloidosis, even in pretreated patients [20,23-25]. However, the data regarding the toxicity and efficacy of BD chemotherapy prior to HDM/SCT are limited for patients with AL amyloidosis. Thus, to determine whether induction therapy with BD is advantageous in patients with AL amyloidosis, we prospectively evaluated a therapeutic regimen consisting of two cycles of BD chemotherapy followed by HDM/SCT in a single-center study.

## Methods

### Patient eligibility

Patients with newly diagnosed AL amyloidosis were enrolled in this trial, which was approved by the institutional ethical review board of Jinling Hospital. The participants or their guardians provided written informed consent. All patients had amyloid disease, which was confirmed by renal biopsy and documented plasma cell dyscrasia; the AL amyloidosis diagnosis and the assessment of organ involvement were based on consensus criteria [26]. Patients who met the three criteria for multiple myeloma (MM) diagnosis, that is, clonal bone marrow plasma cells ≥10%, the presence of serum and/or urinary monoclonal protein and evidence of end-organ damage that can be attributed to the underlying plasma cell proliferative disorder, were excluded [27]. The following HDM/SCT inclusion criteria were applied: age between 18 and 65 years, performance status of 0 to 2 according to Eastern Cooperative Oncology Group (ECOG) criteria [28], a left ventricular ejection fraction (LVEF) >45%, a serum bilirubin level ≤2.0 mg/dl, a pulmonary diffusion capacity ≥50% and a serum creatinine level ≤2 mg/dl. Patients were excluded if they had uncompensated congestive heart failure, symptomatic cardiac arrhythmia, or cardiac syncope.

### Study design

**Figure 1:**
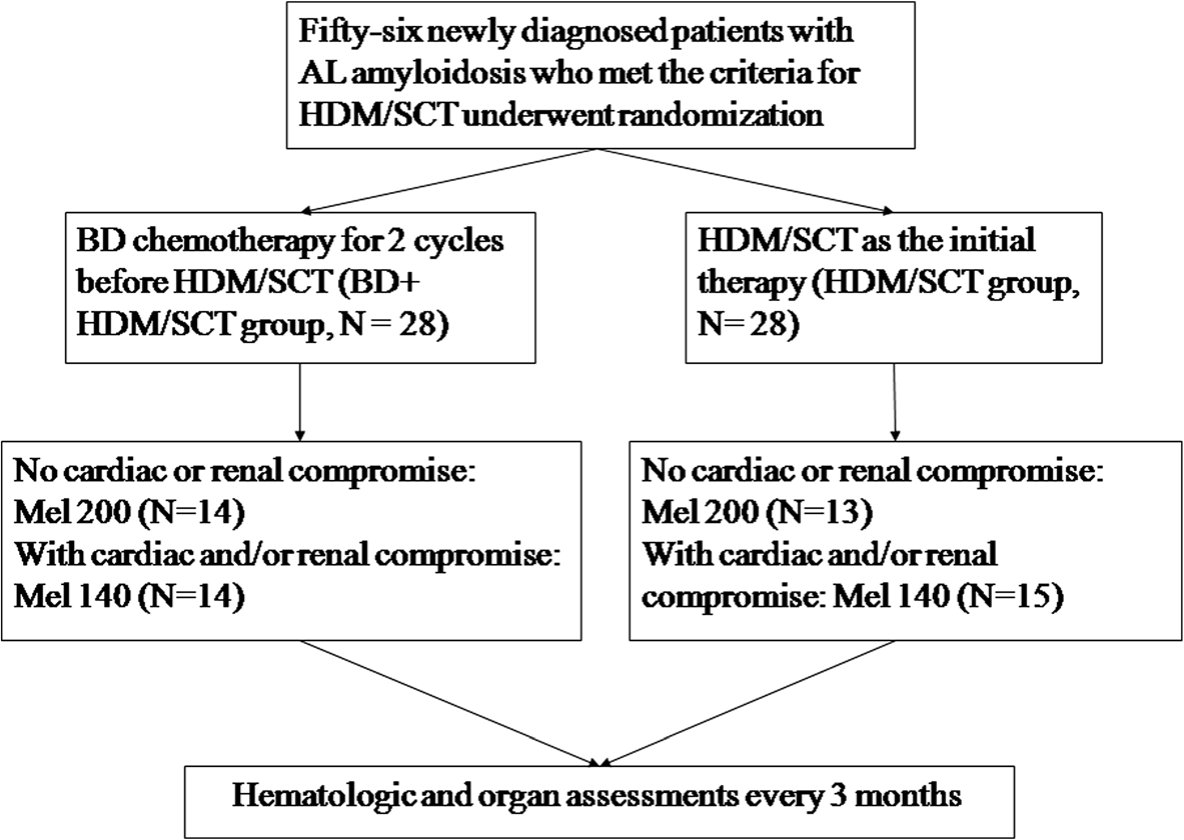
Study schema.

In this prospective, randomized controlled study, newly diagnosed AL amyloidosis patients who met the criteria for HDM/SCT were randomized to receive two cycles of BD as induction therapy followed by HDM/SCT (BD + HDM/SCT) or to receive HDM/SCT alone as an initial treatment. The BD regimen included bortezomib 1.3 mg/m^2^ i.v. and dexamethasone 40 mg p.o. on days 1, 4, 8 and 11 of the 21 day cycle. This process was repeated for two cycles, after which the patients underwent HDM/SCT treatment within eight weeks. The stem cells were mobilized with granulocyte colony-stimulating factor alone, and a minimum collection of 2 × 10^6^ CD34^+^/kg body weight was required. The patients were assigned to one of two MEL dose levels (200 mg/m^2^ or 140 mg/m^2^) based on age, cardiac involvement and renal function [29] (Figure 1). Supportive therapy for side effect management was administered according to the clinical requirements. Omeprazole was given as prophylaxis to all patients who received BD induction, but they were not treated with anti-virus prophylaxis. The patients were assessed every three months following HDM/SCT until progression or death. Adverse events (AEs) were recorded throughout the study and were graded according to the National Cancer Institute Common Terminology Criteria for Adverse Events, version 3.0.

### Hematologic and organ response criteria

Hematologic and organ responses were evaluated according to the novel criteria of the International Society of Amyloidosis [30,31] . Hematologic complete response (CR) was defined as normalization of the FLC levels and ratio, negative serum and urine immunofixation. A hematologic very good partial response (VGPR) was defined as a reduction in dFLCs (difference between involved FLCs and uninvolved FLCs) to <40 mg/L. A hematologic partial response (PR) was defined as a greater than 50% reduction in dFLCs. No response (NR) was defined as less than PR. A hematologic progression has three situations: from CR, any detectable monoclonal protein or abnormal FLC ratio (the light chain must double); from PR, a 50% increase in serum M protein to >0.5 g/dl or 50% increase in urine M protein to >200 mg/day (a visible peak must be present); and an FLC increase of 50% to >100 mg/l. The hematological and organ responses were assessed every three months following HDM/SCT.

### Statistical analysis

The protocol was designed as a superiority trial to demonstrate that BD induction followed by HDM/SCT is superior to HDM/SCT alone for treating AL amyloidosis. The working hypothesis was that the BD induction, followed by the HDM/SCT, would improve the hematologic CR rate (estimated to be 30% in the group assigned to receive the HDM/SCT alone) by 40% at 12 months. The sample size necessary to detect a significant difference (α = 0.05, 2-sided) was calculated to be 23 on the basis of 0.8 power. To compensate for the non-assessable patients, we planned to enroll a minimum of 28 patients per group.

The primary end point for this study was a hematologic CR rate 12 months after HDM/SCT. The secondary end points included the organ response rate, overall survival (OS), and progression free survival (PFS) for all patients. OS was defined as the time from the randomization to the date of death from any cause. The survival time was censored at the date of the last contact for the patients who were still alive or lost to follow-up. PFS was defined as the time from the randomization until date of progression, death, or last follow-up. The PFS and OS between the groups were compared using the Kaplan-Meier method. The start date was the date of randomization, and the cutoff date was June 30, 2013.

The t-test for independent samples or the Mann–Whitney U test was used to compare the continuous data between the groups. The differences between the categorical variables were assessed using Fisher’s exact test. Cox proportional hazards were used to calculate the hazard ratios (HRs) for each variable. The *P* value reported was 2-sided, and *P* <0.05 was considered to be statistically significant. All analyses were performed using SPSS software (version 13.0, SPSS Inc., Chicago, IL, USA).

## Results

### Patient characteristics

Between June 2009 and June 2012, 56 patients (Table 1) with newly diagnosed, treatment-naive AL amyloidosis who provided informed consent were enrolled and treated in this clinical trial. A total of 28 patients were treated with BD induction followed by HDM/SCT (BD + HDM/SCT), while another 28 patients received only HDM/SCT. The median ages and gender compositions of the patients in the two groups were similar. The median time from diagnosis to transplantation was four months (range, 2 to 16 months) in the BD + HDM/SCT group and two months (range, 1 to 18 months) in the HDM/SCT group (*P* = 0.07). The frequencies of systemic involvement were similar in both groups, and all patients presented with renal involvement, as confirmed by renal biopsy. A total of 17 patients (60.7%) in the BD + HDM/SCT group and 16 patients (57.1%) in the HDM/SCT group had involvement of more than one organ. The proportion of heart involvement in the BD + HDM/SCT and HDM/SCT group was 60.7% and 53.5%, respectively; using biomarker cardiac staging criteria [32], the proportion of stage I, II, and III patients in the two groups were 50% versus 42.9%, 35.7% versus 39.3%, and 14.3% versus 17.8%, respectively. The laboratory findings for both groups demonstrated that nephrotic syndrome was the main clinical feature presented by all patients, and that there were no differences between the two groups at the time of diagnosis. BD induction therapy has been reported to quickly reduce the levels of involved FLCs (iFLCs) and dFLCs. Accordingly, the median values of iFLCs and dFLCs in the BD + HDM/SCT group had decreased by more than 50% prior to HDM/SCT.

**Table 1:** Patient characteristics

**Characteristic**	**BD + HDM/SCT (number = 28)**	**HDM/SCT (number = 28)**	***P *** **value**
Age (years)			
Median	53	51.5	0.23
Range	38-65	37-63	
Male/female (number)	18/10	16/12	0.78
Organ involvement, number (%)			
Kidney	28 (100)	28 (100)	1.0
Heart	17 (60.7)	15 (53.5)	0.79
Liver	1 (3.6)	3 (10.7)	0.61
Nervous system	3 (10.7)	2 (7.1)	1.0
>1 organ involved	17 (60.7)	16 (57.1)	1.0
Cardiac stage, number (%)^a^			
I	14 (50)	12 (42.9)	0.86
II	10 (35.7)	11 (39.3)	
III	4 (14.3)	5 (17.8)	
Involved FLC, number (%)			
κ	3 (10.7)	2 (7.1)	1.0
λ	25 (89.3)	26 (92.9)	
Abnormal FLC κ-to-λ ratio number (%)	26 (92.9)	27 (96.4)	1.0
ECOG PS (0/1/2), number (%)			
0	13 (44.4)	14 (46.2)	0.75
1	10 (37)	11 (42.3)	
2	5 (18.6)	3 (11.5)	
24-hour urine protein (g/24 hour)	4.6 ± 2.2	5.8 ± 4.1	0.18
Albumin (g/L)	26.8 ± 6.1	25.7 ± 4.9	0.44
Creatinine (mg/dL)	0.8 ± 0.3	0.8 ± 0.4	0.72
GFR (mL/min)	89.0 ± 19.6	85.1 ± 25.6	0.53
Marrow plasma cells (%)	3.2 ± 2.7	2.8 ± 2.1	0.59
Echocardiogram septal thickness (mm)	11.7 ± 2.4	11.1 ± 1.8	0.33
Ejection fraction (%)	60.8 ± 6.5	62.7 ± 6.1	0.27
Alkaline phosphatasea (U/L)			
Median	58	49	0.11
Range	23 to 321	33 to 661	
NT-proBNP (ng/L)			
Median	268	249	0.32
Range	55.5 to 13,730	15.2 to 7,709	
>8500 ng/L (%)	1 (3.6)	1 (3.6)	
BNP (ng/L)			
Median	117	120	0.85
Range	22 to 3,261	17 to 3,078	
Troponin-I (ng/ml)			
Median	0.003	0.003	0.83
Range	0 to 0.15	0 to 0.12	
iFLC (mg/L)			
Median	114.9	97.8	0.15
Range	25 to 516	24 to 204	
dFLC (mg/L)			
Median	96.1	64.8	0,12
Range	14 to 471	14 to 163	
>180 mg/L (%)	2 (7.1)	0	

^a^The upper reference limit for alkaline phosphatase is 172 U/L. BNP, brain natriuretic peptide; ECOG PS, Eastern Cooperative Oncology Group performance status; FLC*,* free light chain; GFR, glomerular filtration rate.

### Treatment

Of the 28 patients who underwent BD induction, 24 completed the treatment in accordance with the standard dose; four patients developed neuropathy and one patient experienced thrombocytopenia resulting in a dose reduction of bortezomib in the second cycle. Five patients developed grade 3 edema, resulting in a dose reduction of dexamethasone to 20 mg. A total of 14 patients in the BD + HDM/SCT group and 13 patients in the HDM/SCT group received 200 mg/m^2^ MEL whereas the other patients received 140 mg/m^2^ MEL (Figure 1). The median number of stem cells collected was 5.5 × 10^6^ CD34^+^/kg (range: 2 to 13.7 × 10^6^ CD34^+^/kg) in the BD + HDM/SCT group and 3.5 × 10^6^ CD34^+^/kg (range: 2.0 to 14.2 × 10^6^CD34^+^/kg) in the HDM/SCT group (*P* = 0.13). The median granulocyte and platelet engraftment was 10 days and 13 days, respectively.

### Hematologic and organ responses

The hematologic responses and organ response rate are summarized in Table 2. The hematologic overall response rate (ORR) between the BD + HDM/SCT arm and HDM/SCT arm at three, six and twelve months was 78.5% versus 50%, 82.1% versus 53.5% and 85.7% versus 53.5%, respectively, and the CR at three, six and twelve months was 53.6% versus 21.4%, 60.7% versus 28.5% and 67.9% versus 35.7%, respectively. The intention-to-treat (ITT) response rates calculated at 24 months in 20 and 23 patients for the BD + HDM/SCT and HDM/SCT arm were 80% and 47.8%, respectively. There were significant differences in hematologic CR among the patients who were evaluated a year after completing transplantation in the BD + HDM/SCT group and the HDM/SCT group, at 67.9% in the former and 35.7% in the latter (*P* = 0.02). The 12-month ORRs were also higher in the BD + HDM/SCT group (85.7% versus 53.6%, *P* = 0.04), and this group also had higher CR rates at 3, 6 and 24 months. No significant difference was observed between the two groups with respect to the VGPR and PR and the disease progression (PD) rates. A total of 15 patients achieved hematologic response after induction therapy in the BD + HDM/SCT group, of whom eleven patients (39.2%) achieved CR, two achieved VGPR, and two achieved PR. ORR after BD and prior to HDM/SCT in the BD + HDM/SCT arm was 53.5%; seven of the other thirteen BD + HDM/SCT patients who exhibited NR to induction therapy achieved hematologic response at three months post-transplantation (two patients achieved CR, two patients achieved VGPR, and three patients achieved PR).

**Table 2:** Hematological and organ responses

**ITT**	**Months post HDM/SCT**					
	**3**	**3**	**12**	**12**	**24**	**24**
	**BD + HDM/SCT**	**HDM/SCT**	**BD + HDM/SCT**	**HDM/SCT**	**BD + HDM/SCT**	**HDM/SCT**
	**Number = 28**	**Number = 28**	**Number = 28**	**Number = 28**	**Number = 20**	**Number = 23**
CR	15 (53.6%)^a^	6 (21.4%)	19 (67.9%)^a^	10 (35.7%)	14 (70%)^a^	8 (34.8%)
VGPR	4 (14.3%)	4 (14.3%)	2 (7.1%)	3 (10.7%)	1 (5%)	1(4.3%)
PR	3 (10.7%)	4 (14.3%)	3 (10.7%)	2(2.1%)	1 (5%)	2 (8.7%)
NR	6 (21.4%)	12 (42.8%)	2 (7.1%)	6 (21.4%)	2 (10%)	3 (13.0%)
PD	-	-	2 (7.1%)	3 (10.7%)	1 (5%)	3 (13.0%)
Organ responses						
Kidney^b^			65.2% (15/23)	39.1% (9/23)	75% (12/16)	53.8% (7/13)
Heart^b^			67% (10/15)	25% (4/12)	70% (7/10)	50% (3/6)
Liver^b^			100% (1/1)	50% (1/2)	100% (1/1)	100% (1/1)
NS^b^			100% (3/3)	100% (2/2)	100% (3/3)	100% (2/2)

^a^Compared with the HDM/SCT group, *P* <0.05; ^b^evaluable patients. CR, complete hematological response; ITT, intention-to-treat; NR, no response; NS, nervous system; PD*,* disease progression; PR, partial response; SD, stable disease; VGPR, very good partial response.

The ITT analysis of organ response (OR) between the two groups is also summarized in Table 2. A total of 65.2% (n = 15) of the patients in the BD + HDM/SCT group and 39.1% (n = 9) of patients in the HDM/SCT group experienced improvements in at least one involved organ by twelve months. When assessed by individual organ, the response rates of the kidneys and hearts in the BD + HDM/SCT patients were higher than those of the HDM/SCT patients. However, the response rates of the liver and nervous system were similar between the two groups. In both groups, the OR rates of the surviving patients gradually increased with follow-up time.

### Survival and progression

**Figure 2:**
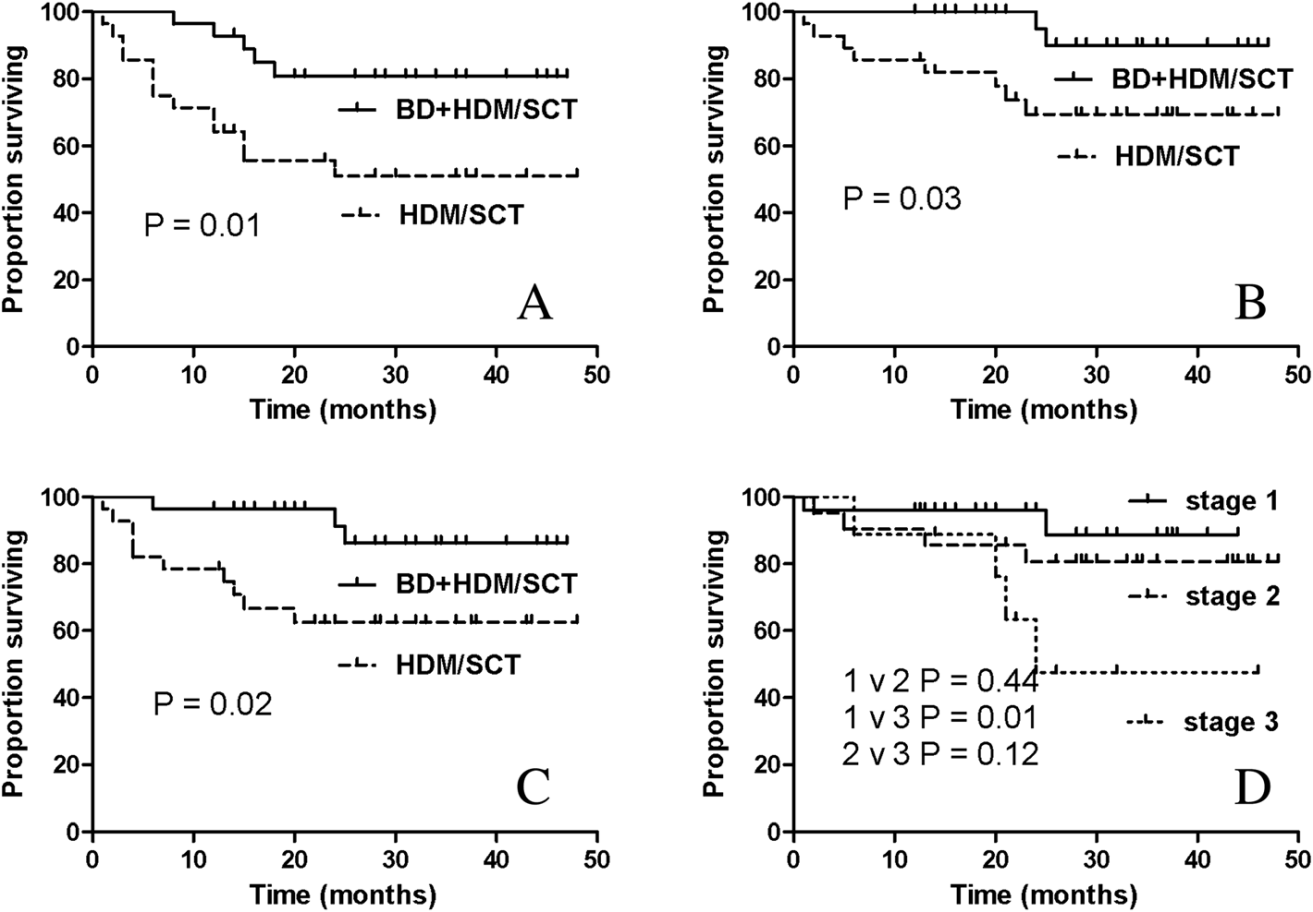
**Progression free survival and overall survival. (A)** The PFS of the two groups; **(B)** the OS of the two groups; **(C)** the renal survival rates of the two groups; and **(D)** The OS of all patients based on the Mayo cardiac staging system.

The median follow-up of the surviving patients was 28 months (range 12 to 8 months), and the data collection for the median PFS and OS is ongoing. The Kaplan-Meier curves for the PFS and OS are shown in Figure 2. Eight patients (two patients from the BD + HDM/SCT group and six patients from the HDM/SCT group) died during the follow-up period. One of these patients died from gastrointestinal bleeding, one died from sepsis, and the others died because of complications related to the progression of the disease. All patients who achieved hematologic CR survived. Three patients in the BD + HDM/SCT group and six patients in the HDM/SCT group had hematologic progression during the follow-up period. Two BD + HDM/SCT patients and three HDM/SCT patients received second-line treatment based on bortezomib or thalidomide, but only one of these patients had a hematologic (partial) response.

PFS and OS differed significantly between the two groups (Figure 2A and B). The patients in the BD + HDM/SCT group had better PFS and OS rates than the patients in the HDM/SCT group. The estimated OS of the patients at 24 months was 95.0% in the BD + HDM/SCT group and 69.4% in the HDM/SCT group (*P* = 0.03); the estimated PFS of patients at 24 months was 80.7% in the BD + HDM/SCT group and 51.1% in the HDM/SCT group (*P* = 0.01). The renal survival (Figure 2C) at 24 months was 91.4% in the BD + HDM/SCT group and 62.6% in the HDM/SCT group (*P* = 0.02). Based on the Mayo cardiac staging system, those patients with stage III cardiac involvement had the worst OS rate (Figure 2D), and the median survival was 24 months. In a multivariate analysis, only troponin-I (HR, 6.138; 95% confidence interval (CI), 1.559 to 24.170; *P* = 0.009) was independently associated with survival (Table 3).

**Table 3:** Factors associated with survival of all patients

**Factors**	**Univariate**			**Multivariate**		
	**HR**	**95% CI**	***P***	**HR**	**95% CI**	***P***
Without BD induction	4.730	1.003-22.307	0.050	4.359	0.913-20.823	0.065
Urine protein >3.5 g/24 hour	1.2	0.310-4.647	0.792			
Cardiac stage III	4.118	1.138-14.899	0.031	1.272	0.276-5.864	0.758
Baseline BNP >170 ng/L	2.837	0.814-9.892	0.104			
Troponin-I >0.03	6.494	1.673-25.213	0.007	6.138	1.559-24.170	0.009
The use of MEL 140	3.445	0.730-16.255	0.118			

BD, bortezomib in combination wity dexamethasone; BNP, brain natriuretic peptide; CI, confidence interval; HR, hazard ratio; MEL, melphalan.

### Treatment-related mortality and toxicity

In total, two patients died within 100 days post-transplantation, resulting in a TRM of 3.6%. Both patients were in the HDM/SCT group: one death was cardiac related, occurring at +60 days, and the other death was caused by hepatic rupture, which occurred at +21 days. The treatment-related toxicities were similar between the two groups (Table 4), with gastrointestinal toxicity as the most common AE. The grade 3 and 4 AEs that were possibly related to BD induction are shown in Table 5. The hematological adverse events (grade 3 and 4) included 34% thrombocytopenia, 15% anemia and 7% neutropenia. The most common non-hematologic BD toxicity was infection (n = 8), while herpes zoster infection was the most common type (n = 5). Edema was also a common toxicity during BD treatment: 17.9% (n = 5) patients developed grade 3 edema.

**Table 4:** Adverse events possibly related to HDM/SCT (Grade >2)

**Toxicity**	**BD + HDM/SCT (n = 28)**	**HDM/SCT (n = 28)**	***P *** **value**
Nausea or vomiting	8 (28.6%)	10 (35.7%)	0.78
Diarrhea	6 (21.4%)	7 (25%)	1.0
Mucositis	5 (17.9%)	7 (25%)	0.75
Hepatic	5 (17.9%)	5 (17.9%)	1.0
Renal	4 (14.3%)	6 (21.4%)	0.73
Cardiac	3 (10.7%)	6 (21.4%)	0.47
Febrile neutropenia	9 (32.1%)	7 (25%)	0.77
Sepsis	0	2 (7.1%)	0.5

BD, bortezomib in combination with dexamethasone; HDM/SCT, high dose melphalan and autologous stem cell transplantation.

**Table 5:** Adverse events possibly related to BD induction

**Toxicity**	**Grade 3**	**Grade 4**	**Grade 5**
Thrombocytopenia, number (%)	8 (28.6%)	1 (3.6%)	0
Neutropenia, number (%)	2 (7.1%)	0	0
Anemia, number (%)	4 (14.3%)	0	0
Gastrointestinal, number (%)	7 (25%)	0	0
Cardiac, number (%)	3 (10.7%)	0	0
Infection, number (%)	8 (28.6%)	0	0
Acute kidney injury, number (%)	2 (7.1%)	0	0
Hepatic, number (%)	3 (10.7%)	0	0
Neuropathy	5 (17.9%)	0	0

## Discussion

The HDM/SCT procedure for AL amyloidosis was introduced in 1998 by Comenzo *et al*., and represented a major breakthrough for AL amyloidosis [33]. Indeed accumulating data have indicated that HDM/SCT can suppress underlying monoclonal plasma cell disease and improve the patient’s quality of life [34]. The purpose of induction chemotherapy is to reduce the light chain load and to improve organ function prior to HDM/SCT. Amyloidosis is characterized by a relatively small tumor mass, so small that induction chemotherapy is often not necessary before proceeding directly to high-dose therapy. To date, the use of induction chemotherapy prior to HDM/SCT has been evaluated in a few studies, which have reported negative results. Pretreatment with two cycles of oral melphalan/prednisone prior to the transplantation did not improve the results of a prospective randomized trial [35]. Similarly, the data from Perz *et al*. [36] indicated that administering vincristine, doxorubicin and dexamethasone (VAD) before HDM/SCT did not increase the hematologic response rate. Nonetheless, the outcome of BD induction prior to HDM/SCT has remained unknown.

Several studies have reported the results of adjuvant BD combination with HDM/SCT, and the addition of bortezomib to the HDM used as a conditioning regimen prior to stem-cell transplantation is feasible and well-tolerated by patients with AL amyloidosis [37]. The BD regimen has also been used following HDM/SCT to improve the depth of the response for patients who achieve less than VGPR. Nineteen of 28 patients received post-transplant BD chemotherapy, with 67% of these achieving CR and 60% organ responses [38]. A complementary approach of administering two cycles of BD prior to and as conditioning for HDM/SCT also yielded very good response rates of hematological remission in 9/18 patients after the induction, and all 11 patients evaluable after HDM/SCT attained CR/VGPR [39]. The combination of BD with cyclophosphamide has also shown encouraging response rates in patients with AL amyloidosis, even without high-dose therapy [40,41]. Additionally, most of the patients in these two series were either transplant ineligible or relapsed. Therefore, based on the current knowledge regarding the use of BD in the treatment of AL amyloidosis, it is clear that the BD regimen is effective and fast acting in patients with AL amyloidosis. Our data also show that BD therapy prior to HDM/SCT can quickly reduce the FLC level and the tumor burden and can furthermore delay the progress of involved organs, thus improving the safety and efficiency of HDM/SCT. Therefore, the BD regimen is an appropriate therapy before HDM/SCT. For patients who have already achieved CR prior to HDM/SCT, it is difficult to assess whether HDM/SCT was necessary, and only a clinical trial can provide an answer. Regardless, we believe that additional therapy with HDM/SCT may be able to improve the depth of the response and further extend PFS of those patients.

Our data demonstrate that BD induction followed by risk-adapted HDM/SCT is a more effective strategy than HDM/SCT alone for treating newly diagnosed patients with AL amyloidosis. The majority of patients (85.7%) in the BD induction group achieved hematological responses, including more than half (67.9%) who achieved CR at one year post-therapy. The BD induction group also displayed better PFS and OS rates than the group that received HDM/SCT alone. HDM/SCT treatment in patients with cardiac involvement remains challenging, particularly for patients with stage III cardiac involvement. In our series, 44.4% (4/9) of the patients with stage III cardiac involvement died during follow-up, but the median survival time was more favorable than previously reported [32].

The disadvantages of induction chemotherapy in patients with AL amyloidosis include the potential risk of side effects, the risk of further worsening organ function and the consequent delay of HDM/SCT. Our data demonstrate that the majority of patients can tolerate two cycles of BD treatment, and that the toxicity of the BD regimen is moderate and manageable. All toxicities were reversible and did not influence the subsequent HDM/SCT procedure because MEL toxicity differs from that of bortezomib. However, additional attention should be given to some toxicities that can result in bortezomib or dexamethasone dose reduction, including edema, peripheral neuropathy, and thrombocytopenia. Edema is a common toxicity for high-dose dexamethasone, particularly in patients with renal involvement and low serum albumin levels. Because the serum albumin levels of all five patients who developed grade 3 edema were lower than 2 g/dl, support treatment with diuretics and serum albumin is important for these patients. Peripheral neuropathy also needs to be carefully monitored during BD induction, as 18% of the patients developed grade 3 neuropathy in this study, apparently higher than in other studies [24,25]. One study demonstrated that 1.6 mg/m^2^ once-weekly bortezomib dosing can reduce toxicity compared to 1.3 mg/m^2^ twice-weekly dosing [42]. Subcutaneous bortezomib is another option to reduce the incidence of peripheral neuropathy and has been confirmed in a randomized, phase 3 study for multiple myeloma patients [43] and was also proven in a small series of AL amyloidosis patients [44]. In addition, we observed a high incidence of herpes zoster infection during BD induction; thus, anti-virus prophylaxis appears to be necessary for these patients. In our study, the occurrence of TRM in our study population of 56 patients was 3.6% (2/56), a lower value than in most reports. Moreover, no patients in the BD induction group died because of treatment-related complications. The possible reasons for the relatively low TRM observed include the risk-adapted approach of HDM/SCT, the induction chemotherapy used in half of the patients, the small proportion of patients with severe cardiac involvement and racial differences.

This study has several limitations. First, all of the patients had renal involvement, which most likely constitutes a selection bias because the study was conducted in a nephrology institution. Second, neither group met the median PFS and OS rates, and the long-term patient prognosis requires further study. Third, it is unclear whether the PFS rate of the patients who achieved CR after the BD induction following HDM/SCT is equivalent to the CR achieved with BD therapy alone. Fourth, the sample size of this trial is too small to allow patient stratification based on known risk factors, as well as to allow a subgroup analysis, so the results for stratification or subgroup analysis cannot match the results of other studies. We will continue this study and enroll more patients to make this stratification or subgroup analysis more clear. Additionally, the appropriate salvage regimen for patients who had no response to BD induction following HDM/SCT is also unclear.

## Conclusions

In conclusion, our preliminary data suggest that induction therapy with BD followed by HDM/SCT is an effective and tolerable regimen for treating patients with AL amyloidosis. This protocol can significantly improve both the hematological and organ response rates, and the risk of the BD + HDM/SCT regimen is apparently comparable to that of HDM/SCT. Although HDM/SCT remains a high-risk treatment modality for AL amyloidosis, new agents, such as bortezomib, may alter this therapeutic approach and improve patient outcomes. Further study will be required to establish the long-term benefits of this treatment.

## Abbreviations

AEs: adverse events; BD: bortezomib in combination with dexamethasone; BNP: brain natriuretic protein; CR: complete response; ECOG: Eastern Cooperative Oncology Group; FLC: free light chains; HDM/SCT: high dose melphalan and autologous stem cell transplantation; HRs: hazard ratios; ITT: intention to treat; MEL: melphalan; NR: no response; OR: organ response; ORR: overall response rate; OS: overall survival; PD: disease progression; PFS: progression free survival; PR: partial response; TRM: treatment-related mortality; VAD: vincristine doxorubicin and dexamethasone; VGPR: very good partial response.

## Competing interests

The authors declare that they have no competing interests.

## Authors’ contributions

ZL, XH and QW designed and performed the research, analyzed the data and wrote the paper. WC performed research and analyzed data. XH, QW, WC, CZ, ZC, DG, HZ and ZL took care of the patients. All of the authors read and approved the final manuscript.

## Pre-publication history

The pre-publication history for this paper can be accessed here:

http://www.biomedcentral.com/1741-7015/12/2/prepub
